# Erratum

**DOI:** 10.1080/23802359.2016.1184422

**Published:** 2016-06-16

*Mitochondrial DNA Part B*, issue 1-01 http://www.tandfonline.com/toc/tmdn20/1/1

When the initial articles forming part of this issue were first published online, their pagination was incorrect. All articles up to, and including, Yu P, Kong L, Li Y, Cong H, Li Y. 2016. Analysis of complete mitochondrial genome and its application to phylogeny of *Caryomys inez* (Rodentia: Cricetidae: Arvicolinae), have now been repaginated and republished online.

Taylor & Francis apologises for this error.

